# Vestibular Aqueduct Hypoplasia Identifies Semicircular Canal Dehiscence Patients Who Progress to Meniere’s Disease

**DOI:** 10.1097/ONO.0000000000000079

**Published:** 2025-11-06

**Authors:** David Bächinger, Ann Sophie Franzen, Judith S. Kempfle, Adrian Dalbert, Julia Dlugaiczyk, Tobias Kleinjung, Amy Juliano, Daniel J. Lee, Andreas H. Eckhard

**Affiliations:** 1 Department of Otorhinolaryngology, Head and Neck Surgery, University Hospital Zurich, Zurich, Switzerland; 2 University of Zurich, Zurich, Switzerland; 3 Eaton-Peabody Laboratories, Mass Eye and Ear, Boston, MA; 4 Department of Otolaryngology-Head and Neck Surgery, Harvard Medical School, Boston, MA; 5 Department of Radiology, Mass Eye and Ear, Harvard Medical School, Boston, MA; 6 Otopathology Laboratory, Mass Eye and Ear, Boston, MA.

**Keywords:** Endolymphatic sac, Meniere’s disease, Temporal bone development, Vestibular aqueduct

## Abstract

**Objective:**

Semicircular canal dehiscence (SCD) and vestibular aqueduct (VA) hypoplasia are developmental anomalies associated with distinct inner ear syndromes—SCD syndrome and Meniere’s disease (MD). Our previous work found frequent SCD in MD patients with VA hypoplasia, suggesting a shared developmental origin. To further explore this association, we adopted a reciprocal approach by assessing VA hypoplasia prevalence in patients diagnosed with SCD.

**Study Design:**

Retrospective cohort study.

**Setting:**

Tertiary referral center.

**Patients:**

A total of 219 ears from 173 patients (mean age 53.5 years, standard deviation 16.6 years; 54.3% females) were evaluated for suspected SCD, confirmed by temporal bone computed tomography (CT).

**Interventions:**

Radiological analysis of temporal bone CT scans using the angular trajectory of the vestibular aqueduct (ATVA) marker; review of clinical records for diagnosis of MD.

**Main Outcome Measures:**

Prevalence of VA hypoplasia among SCD patients; association of radiological findings with clinical diagnosis of MD.

**Results:**

VA hypoplasia was identified in 4 of 173 patients (2.3%), representing a 46-fold increase compared with the estimated 0.05% prevalence of MD patients with VA hypoplasia in the general population. These cases showed an atypical SCD localization in the posterior limb of the superior semicircular canal. All 4 patients were diagnosed with MD ipsilateral to the SCD/VA hypoplasia.

**Conclusions:**

SCD and VA hypoplasia are associated in a distinct patient group, suggesting a shared developmental etiology. These patients may be predisposed to an early overlap of SCD syndrome and MD, with their clinical course eventually dominated by the progressive nature of MD.

Semicircular canal dehiscence (SCD) is an abnormal opening in the bony shell (otic capsule) of a semicircular canal (SCC), creating a “third window”—an aberrant pathway allowing pressure wave transmission between the cerebrospinal fluid and the inner ear’s perilymphatic fluid space (1). Although often detected incidentally on imaging in asymptomatic individuals, approximately 20% of SCD cases (2–4) manifest with distinct auditory and vestibular symptoms, that is, SCD syndrome (5). SCD is generally considered a developmental anomaly of hypoplastic bone growth/incomplete ossification of the otic capsule (6–11), though alternative mechanisms such as successive bone thinning/erosion have also been proposed (2,12,13), and the two processes likely occur in different patient populations (2).

Our previous work demonstrated that approximately 30% of patients with Meniere’s disease (MD) exhibit vestibular aqueduct (VA) hypoplasia, a key pathology in this subgroup termed “MD-hp” (14–16). Notably, these MD-hp patients had a 3- to 8-fold higher prevalence of radiological SCD (29.4%; (16)) compared with MD patients without VA hypoplasia (3.6%; (16)) and the general population (3%–10%; (17–19)). This striking co-occurrence raised the possibility that VA hypoplasia and a subset of SCD may share a common developmental origin, and that both may even cause temporally overlapping clinical symptoms. To further investigate this potential link, we investigated the reverse relationship: assessing the prevalence of VA hypoplasia among patients initially diagnosed with radiological SCD, with or without clinical SCD syndrome.

## MATERIALS AND METHODS

### Ethical Considerations

This retrospective study was approved by the local Institutional Review Board and was conducted in accordance with the Declaration of Helsinki and its amendments.

### Study Population

We retrospectively reviewed a cohort of consecutively assessed patients who attended the neurotology clinic at a tertiary referral center. Inclusion criteria required omputed tomography (CT)-confirmed SCD, as diagnosed by an experienced neuroradiologist. Patients were included regardless of whether they exhibited symptoms consistent with SCD syndrome (20). Patients were excluded if the radiology report described the SCC bone as “markedly thinned” or “papyraceous,” but not frankly dehiscent or with a defect. Clinical records and radiology reports were examined to document diagnoses of SCD syndrome and any concomitant MD diagnosis in all patients (21,22). Due to the retrospective study design, documentation of the clinical reasoning for initial SCD evaluation was not consistently available.

### Temporal Bone CT Imaging

High-resolution multidetector CT (slice thickness: 0.625 mm) or cone-beam CT (slice thickness: 0.5 mm) imaging of the temporal bones was performed without intravenous contrast using standard clinical protocols. Images were reconstructed separately for each temporal bone in the axial, coronal, Pöschl, and Stenvers planes using a standard bone algorithm.

### Angular Trajectory of the Vestibular Aqueduct

The angular trajectory of the vestibular aqueduct (ATVA) was determined on CT images (reformatted axial in the plane of the horizontal SCC) following established methods (15,16,23). Briefly, 2 angles are measured on horizontal sections: the angle representing the trajectory of the proximal VA relative to the medial wall of the vestibule and the angle reflecting the trajectory of the distal VA relative to the proximal VA segment, defining the ATVA. In accordance with prior literature (15), an ATVA bending angle exceeding 140° was considered indicative of a hypoplastic VA (and endolymphatic sac), whereas an angle below 120° was classified as normal (15). All imaging analyses were performed by an investigator (A.S.F.) with no access to clinical patient data. The mean ATVA of the present study’s SCD cohort was compared with a previously characterized control group with no SCD (15).

### Quantitative Analysis of Superior Semicircular Canal Dehiscence

Length of the dehiscence was quantified similarly to previously published methods (24). In brief, SSC reconstructions in the Pöschl plane were generated using Horos (Nimble Co., LLC, Purview, Annapolis, MD). Measurements of the dehiscence length and its distance from the ampulla were performed using Fiji/ImageJ2 (v2.14.0/1.54f (25)).

### Inter-rater Reliability of ATVA Measurements

To evaluate inter-rater reliability, a second blinded investigator (D.B.) independently repeated the ATVA measurements in 44 cases (20.1% of the total). Before measurement, both investigators underwent a supervised training period led by an experienced researcher to ensure consistency in the measurement protocol. Spearman’s rank correlation coefficient and the intra-class correlation coefficient were calculated, yielding a Spearman’s rho of 0.78 (95% confidence interval [CI], 0.60–0.88; *P* < 0.0001) and an intra-class correlation coefficient of 0.76, indicating excellent agreement and data consistency (26), consistent with previous ATVA inter-rater reliability following a supervised training phase (27).

### Statistical Analysis

All statistical tests were prespecified before data collection. Analyses were conducted using IBM SPSS Statistics for Windows (version 25; IBM Corp., Armonk, NY) and GraphPad Prism (version 7; GraphPad Software, La Jolla, CA). A *P* value of less than 0.05 was considered statistically significant. Unless otherwise specified, values are reported as means and standard deviations (SD) or as absolute numbers with percentages.

## RESULTS

### A Subset of SCD Patients Shows Radiographic Evidence of VA hypoplasia

**FIG. 1.**
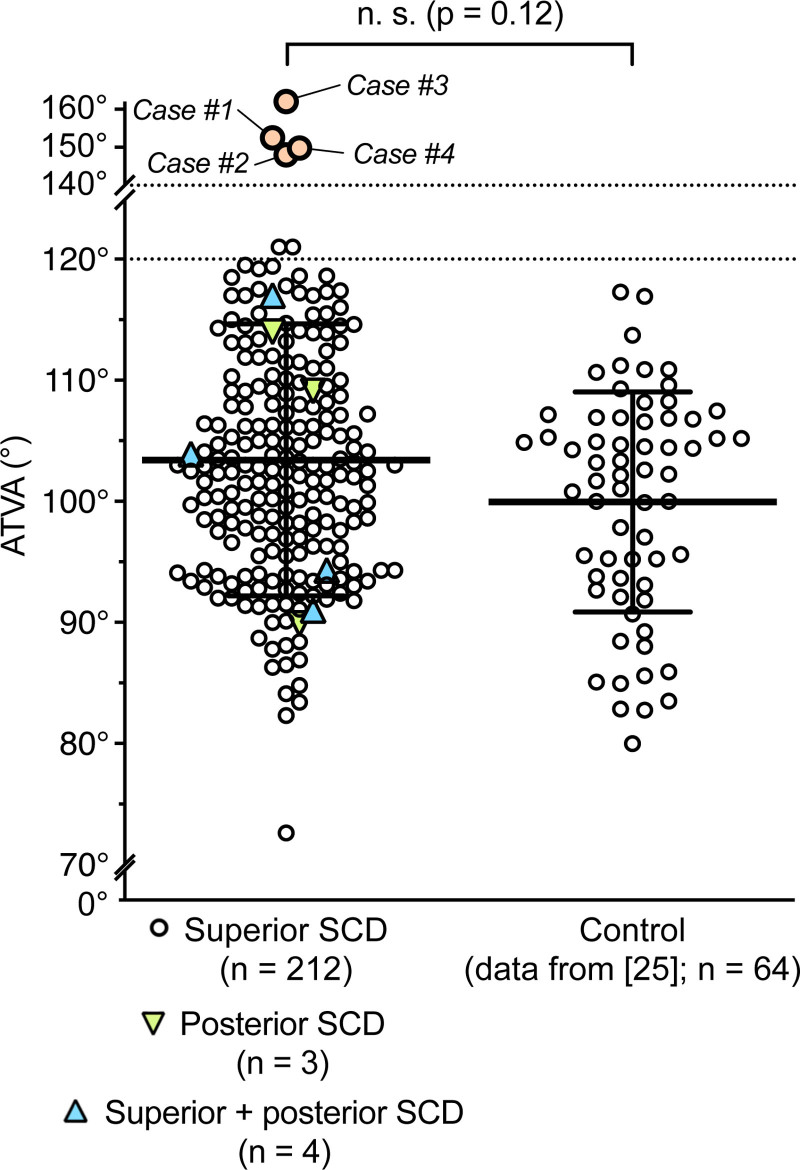
Angular trajectory of the vestibular aqueduct in patients with semicircular canal dehiscence (SCD) compared to a previously published control cohort (15). SCD patients with concomitant Meniere’s symptoms are indicated (cases #1–#4; orange); n.s., not significant; bold horizontal line, mean; whiskers, standard deviation.

We analyzed 219 ears on temporal bone CT scans from 173 patients with radiologically confirmed SCD (Fig. 1). The mean age was 53.5 (SD 16.6) years, with 94 females (54.3%) and 79 males (45.7%). Unilateral SCD was observed in 127 patients (73.4%), and bilateral SCD in 46 patients (26.6%). Superior SCD was the predominant finding, identified in 212 otic capsules from 168 patients (97.1%), followed by posterior SCD in 3 otic capsules from 2 patients (1.2%) and combined superior and posterior SCD in 4 otic capsules from 3 patients (1.7%). Clinically, 116 patients (67.1%) were diagnosed with SCD syndrome, and 4 patients (2.3%) had a concomitant MD diagnosis. The mean ATVA in the SCD cohort was 103.4° ± 11.2°, which is not significantly (*P* = 0.12) different from a previously characterized control cohort without SCD or MD, based on normal temporal bone CT scans (99.9° ± 9.1°). Notably, while most ATVA values were below 120° (indicating normal VA morphology), four ears exhibited ATVA values >140°, consistent with VA hypoplasia (Fig. 1).

### Patients with Both SCD and VA Hypoplasia Progress to Meniere’s Disease

Four patients (cases #1–#4) exhibited both SCD and VA hypoplasia on CT imaging. In cases #1–#3, the ear affected by both anomalies eventually received an MD diagnosis. In case #4, bilateral VA hypoplasia was present alongside a progressive audiovestibular syndrome, strongly suggestive of bilateral MD, although a formal MD diagnosis was not documented in available clinical records.

**FIG. 2.**
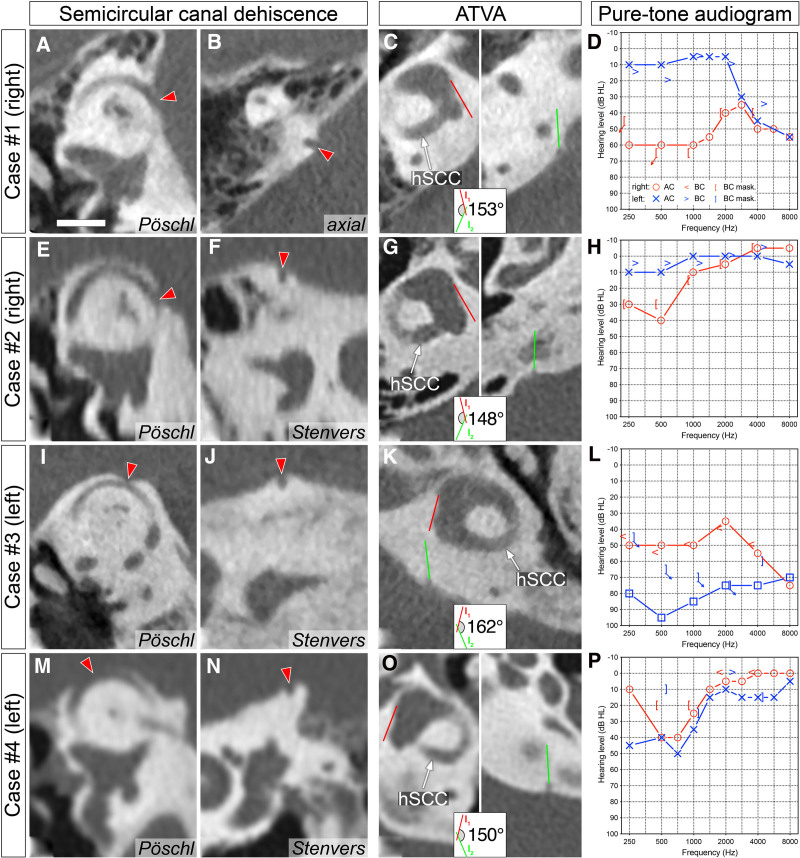
CT imaging and audiometric data from SCD patients with concomitant VA hypoplasia, defined by an ATVA >140°. The first and second columns depict SCD (red arrowheads) in Pöschl and Stenvers/axial planes. The third column illustrates ATVA measurement in the axial plane, defined as the angle formed by the proximal VA at the level of the horizontal semicircular canal (red line) and the distal VA at the level of the operculum (green line), as previously described (15). An angle >140° indicates a hypoplastic VA and endolymphatic sac. The last column presents pure-tone audiometry at the time of Meniere’s disease diagnosis (cases #1–3) or the most recent audiometric evaluation (case #4). Scale bar: 5 mm. ATVA, angular trajectory of the vestibular aqueduct; SCD, semicircular canal dehiscence; VA, vestibular aqueduct.

Case #1: A 66-year-old male exhibited right superior SCD (Fig. 2A–B) and right VA hypoplasia (Fig. 2C). His clinical presentation—including prolonged spinning vertigo lasting several hours, sensorineural hearing loss as confirmed by pure-tone audiometry (Fig. 2D), and right-sided tinnitus—evolved to meet MD criteria (22). The patient did not report any symptoms specifically attributable to SCD syndrome. Vestibular testing revealed right-sided hyporeflexia on caloric examination. Cervical vestibular evoked myogenic potential thresholds were within normal limits bilaterally (90 dB HL on both sides at 500 Hz air-conducted stimulation). Progressive to severe levels of hearing loss ultimately led to right cochlear implantation combined with labyrinthectomy.

Case #2: A 17-year-old male was initially diagnosed with right superior SCD syndrome based on CT findings of superior SCD (Fig. 2E–F) with tinnitus, sensorineural low-frequency hearing loss (Fig. 2H), and disequilibrium. As his symptoms evolved to vertigo episodes of several hours, the diagnosis was revised to MD. CT imaging showed right VA hypoplasia (Fig. 2G).

Case #3: A 62-year-old male demonstrated left superior SCD with concomitant VA hypoplasia in the left as well as in the right ear (Fig. 2I–K). He was eventually diagnosed with bilateral MD based on recurrent vertigo episodes lasting several hours, as well as initially fluctuating, left-dominant sensorineural hearing loss, which was more pronounced in the low frequencies. Over time, the hearing loss progressed to moderate-to-severe levels (Fig. 2L).

Case #4: A 40-year-old female exhibited left superior SCD (Fig. 2M–N) and was found to have left VA hypoplasia (Fig. 2O). Although she was not formally diagnosed with MD, her bilateral sensorineural low-frequency hearing loss (Fig. 2P), episodes of imbalance and self-motion, and worsening hearing thresholds are consistent with bilateral definite MD (22). Additionally, CT imaging revealed VA hypoplasia also in her right ear, further supporting the diagnosis of bilateral MD (28).

### Atypical Posterior SCD Localization in Patients with Concurrent VA Hypoplasia

**FIG. 3.**
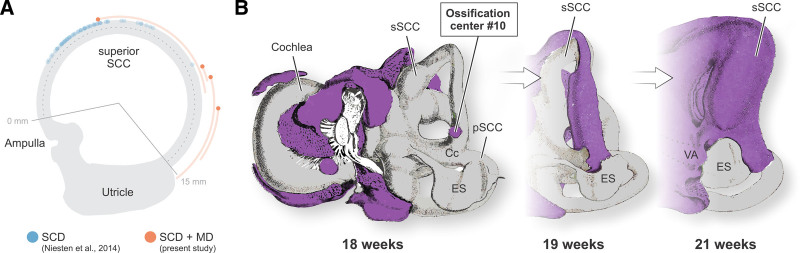
Atypical SCD localization in patients with concurrent VA hypoplasia and its relation to otic capsule development. A, Ampullary border points of SCDs from a previously published cohort (SCD; n = 118; blue) (24) compared to the present cases with concurrent VA hypoplasia and MD (SCD+MD; n = 4; orange). While typical SCDs cluster near the ampulla in the anterior limb of the sSCC, those in VA hypoplasia cases are significantly shifted to the posterior limb (p = 0.02). B, Schematic of otic capsule ossification centers (magenta) surrounding the membranous labyrinth (gray) at 18, 19, and 21 weeks gestation (adapted from (29), which is accessible under the terms of the Creative Commons Zero [CC0] 1.0 license). Ossification center #10, emerging at week 18, contributes to the bony covering of both the posterior sSCC and the VA, suggesting a shared developmental origin for the observed anomalies. Cc, common crus; ES, endolymphatic sac; MD, Meniere’s disease; pSCC, posterior semicircular canal; SCD, semicircular canal dehiscence; sSCC, superior semicircular canal; VA, vestibular aqueduct.

To better understand the spatial relationship between superior SCD and VA hypoplasia, we compared the location of the superior SCD relative to the ampulla in the 4 patients with concurrent VA hypoplasia to a previously characterized SCD cohort (n = 118) (24). In all four cases, the dehiscence was situated significantly farther from the ampulla and consistently localized to the posterior limb of the superior SCC (sSCC)—a pattern distinct from the more typical anterior limb or vertex locations. On average, the distance from the ampulla was 3.4 mm longer than in the control cohort (*P* = 0.02; Fig. 3A). This topographical shift positions the SCD in closer proximity to the hypoplastic VA, suggesting a localized developmental defect as a common origin for both anomalies in this patient subset (Fig. 3A).

## DISCUSSION

Several findings in this exploratory retrospective study collectively suggest a shared developmental etiology of SCD and VA hypoplasia in a subset of patients. First, 2.3% (4 out of 173) of patients with radiologically confirmed SCD had concurrent VA hypoplasia, all of whom eventually developed clinical MD in the affected ear, and with their VA hypoplasia meeting the diagnostic criteria for the hypoplastic endotype of MD (MD-hp) (15). No additional MD cases—that is, without VA hypoplasia—were identified in our cohort. This MD-hp prevalence is 46-fold higher than the expected 0.05% prevalence in the general population (22,23), and 2.5–3 times higher than in general MD cohorts, where the MD-hp subgroup typically represents only about 30–40% of cases overall (14,16). Third, 3 of the 4 identified patients were male, aligning with prior observations of a strong male predominance in MD-hp cohorts (16). Together with our previous reciprocal finding that 29.4% of MD-hp patients had radiological evidence of SCD (16), these results highlight a striking reciprocal clustering of SCD and VA hypoplasia, strongly suggesting a shared developmental pathway.

The 4 patients with concurrent VA hypoplasia all exhibited an atypical localization of their SCD in the posterior limb of the sSCC—rather than in the more commonly affected anterior limb or vertex (Figure 5 in (24))—placing these SCDs in spatial proximity to the (hypoplastic) VA. Notably, the bony covering of both the posterior limb of the superior SCC and the VA originates embryologically from the same “ossification center” (center #10; Figure 3B), 1 of 14 distinct centers that undergo coordinated growth and fusion during fetal development to form the otic capsule (29). This shared developmental origin opens the possibility that a localized defect in this retrolabyrinthine region may underlie the hypoplastic formation of both.

While some genetic studies have proposed an overlapping genetic basis in SCD and MD—particularly pointing to mutations in the cochlin (*COCH*) gene, which plays a role in inner ear structure and maintenance (10,30)—these findings have not been replicated and ultimately remain inconclusive (31). Others have observed and explained the co-occurrence of SCD and endolymphatic hydrops (32) and MD (33), respectively, by the “third window” effect created by the SCD, proposing that the pathological connection between perilymph and the cerebral fluid space effectively lowers perilymph pressure, promoting expansion of the endolymphatic fluid. However, this theory cannot explain why only a subset of SCD patients develop hydrops or MD. Our findings support an alternative model: that VA hypoplasia—an established risk factor for MD (28)—is a prerequisite for hydrops to occur, and in these patients, the underlying developmental abnormality that resulted in VA hypoplasia also resulted in posterior-limb superior SCD. In other words, SCD is not the etiology of MD-hp but is a common coexisting finding among those with MD-hp.

Clinically, the co-occurrence of SCD and VA hypoplasia raises the possibility of a complex and evolving “overlap syndrome,” in which patients may initially exhibit features of both SCD syndrome (eg, sound-induced vertigo, autophony) and early-stage MD (eg, fluctuating hearing loss, episodic vertigo). This concept is further supported by endolymphatic hydrops—a sensitive, albeit nonspecific, finding in MD—being observed in patients with SCD syndrome (34). As MD progresses, its progressive course may ultimately dominate the clinical picture. Our study was not designed to systematically identify such syndromes, given its retrospective nature and limitations in available documentation. Furthermore, the posterior localization of SCD observed in these patients—a region previously associated with milder or atypical audiovestibular symptoms (24)—raises the possibility that some patients may have been experiencing early manifestations of MD already during their initial clinical presentation.

Limitations of our study include its retrospective design and the use of a cohort from a single clinical practice at a tertiary referral center, which may restrict the generalizability of our findings. Furthermore, the retrospective nature of our data did not allow us to capture the nuanced clinical presentations necessary to fully define the proposed overlap syndrome between SCD syndrome and MD in patients with concurrent VA hypoplasia. Prospective studies are warranted to confirm these findings, characterize overlap syndromes, and better understand the timing and progression of symptoms in affected individuals.

In conclusion, our findings suggest that a subset of patients initially evaluated for SCD also exhibit VA hypoplasia and are predisposed to developing MD. These patients may be at risk early in their disease course for a complex overlap syndrome that combines features of both conditions. Early CT-based identification of these concurrent anomalies may help reduce diagnostic uncertainty and guide more proactive, tailored clinical management. Finally, the anatomical proximity of SCD in the posterior limb of the sSCC and hypoplasia of the VA strongly suggest a shared developmental defect in the retrolabyrinthine temporal bone.

## ACKNOWLEDGEMENTS

The authors thank Dr. Steven D. Rauch and Dr. Joe C. Adams (both Massachusetts Eye and Ear, Boston, MA) for critical comments on the manuscript.

## FUNDING SOURCES

D.B. was supported by a national MD-PhD scholarship from the Swiss National Science Foundation (SNSF). A.H.E. was supported by a career development (Filling the gap) grant from the University of Zurich.

## CONFLICTS OF INTEREST

None declared.

## DATA AVAILABILITY STATEMENT

The datasets used and analyzed during the current study are available from the corresponding author upon reasonable request.

## DECLARATION

During the preparation of this work, the authors used Google Gemini and ChatGPT to support the writing process by improving the readability and language of the manuscript. These tools were applied with human oversight and control. The authors carefully reviewed and edited all content.
